# Host developmental stages shape the evolution of a plant RNA virus

**DOI:** 10.1098/rstb.2022.0005

**Published:** 2023-03-27

**Authors:** Izan Melero, Rubén González, Santiago F. Elena

**Affiliations:** ^1^ Instituto de Biología Integrativa de Sistemas (CSIC - Universitat de València), Paterna, 46182 València, Spain; ^2^ The Santa Fe Institute, Santa Fe 87501, NM, USA

**Keywords:** plant–virus interaction, developmental stages, host age, virus evolution

## Abstract

Viruses are obligate pathogens that entirely rely on their hosts to complete their infectious cycle. The outcome of viral infections depends on the status of the host. Host developmental stage is an important but sometimes overlooked factor impacting host–virus interactions. This impact is especially relevant in a context where climate change and human activities are altering plant development. To better understand how different host developmental stages shape virus evolution, we experimentally evolved turnip mosaic virus (TuMV) on *Arabidopsis thaliana* at three different developmental stages: vegetative (juvenile), bolting (transition) and reproductive (mature). After infecting plants with an *Arabidopsis*-naive or an *Arabidopsis*-well-adapted TuMV isolate, we observed that hosts in later developmental stages were prone to faster and more severe infections. This observation was extended to viruses belonging to different genera. Thereafter, we experimentally evolved lineages of the naive and the well-adapted TuMV isolates in plants from each of the three developmental stages. All evolved viruses enhanced their infection traits, but this increase was more intense in viruses evolved in younger hosts. The genomic changes of the evolved viral lineages revealed mutation patterns that strongly depended on the founder viral isolate as well as on the developmental stage of the host wherein the lineages were evolved.

This article is part of the theme issue ‘Infectious disease ecology and evolution in a changing world’.

## 1. Introduction

Viruses are obligate intracellular parasites that hijack their hosts’ cellular components. When a virus takes control of host cells, cellular resources are diverted to generation of viral factories and production of all required viral components, ending in the assembly and liberation of progeny virions. Because of that, the biochemical and physiological status of host cells will strongly impact the ability of a virus to accomplish its replication. This cellular status will depend on multiple and diverse factors: from the host genetics to the environmental conditions the host faces [1]. These internal and external factors drive the host–virus interactions, meaning that different host conditions may result in different susceptibilities to infection.

Host homogeneity is rare in natural populations, which implies that viruses must face host populations whose individuals are genetically diverse and have different degrees of susceptibility to infection [2,3]. The interaction between pathogens and hosts with genetic variability in susceptibility to infection has been well studied [3–5], as well as the influence of developmental stages on hosts' resistance to pathogens [6–9]. Variation in disease susceptibility among hosts of different age or developmental stages constitutes one of the most important components of host heterogeneity potentially affecting the outcome of host–pathogen interactions and their epidemiological dynamics [10]. These differences in susceptibility and resistance might imply that a virus might be adapted to a given host developmental stage and might be unsuccessful infecting others. In nature, an interaction between host developmental stage and susceptibility to infection has been observed in many animal and plant systems for a wide range of pathogens such as bacteria, fungi and viruses. In animals, young individuals are more susceptible to infection than older individuals [11–13]. However, for other pathogens such as fungi, older animals may exhibit increased susceptibility compared with juvenile ones [14]. In plants, a common observation is that hosts become more resistant to pathogens as they mature [15–17]. This is explained by the existence of a plant mechanism known as age-related resistance (ARR), which was first described in the *Arabidopsis thaliana*–*Pseudomonas syringae* pathosystem [18]. ARR is directly dependent on the accumulation of salicylic acid in the intercellular space [19–21], which acts as an antimicrobial agent and accumulates only in old competent ARR plants but not in young susceptible ones. However, some plants can become more susceptible to certain fungal pathogens as they develop [22]. Similarly, recent work by Huang *et al*. [23] has described that older *Arabidopsis* plants are more susceptible to some RNA viruses. Therefore, ARR seems not to be universal and depends on the particular pathosystems being studied.

Owing to their sessile lifestyle, plants need to finely coordinate their growth and development to optimize fitness through rapid and appropriate responses to different stresses they might face. The life cycle of flowering plants can be considered, indeed, as a succession of distinct growth phases. One of these phases is the transition from a juvenile vegetative to a mature reproductive stage. The transition to flowering is under the control of a complex genetic network that integrates information from both endogenous and environmental cues [24], where plants change their genetic programmes to switch from growth to reproduction. Even though flowering is not the developmental transition needed for increased pathogen resistance [25], several studies have described a connection between flowering and defence. Depending on the pathogen or the host genetics, a virus infection can delay reproduction [26,27] or accelerate flowering [28,29]. These strategies can be explained by resource allocation theories, where plants have a limited pool of resources which they must employ to carry out different functions in order to progress and complete their life cycle, therefore promoting trade-offs [30,31]. These decisions happen as plants develop: at a given point, plants switch their resources from biomass accumulation and production of plant structures to reproduction. A similar switch happens when a plant faces a pathogen and needs to strategize and use its resources effectively to overcome the infection. In this sense, a particularly well-studied trade-off is the reallocation of resources from defence to flower development. This reallocation will be particularly convenient for plants that reproduce only once in their lifespan and need to produce descendants at any cost. Furthermore, it has been shown that pathogens face different defence responses depending on the developmental stage of the plant [16,28]. The differences in the response may lead to different pathogen strategies to counteract their hosts’ actions. Indeed, Pagán *et al*. [32] studied host developmental stage and its influence on virus accumulation using *A. thaliana* and cucumber mosaic virus (CMV). They found that for some *Arabidopsis* genotypes, virus accumulation and virulence differed depending on whether plants were inoculated at vegetative or reproductive growth stages. Developmental-stage-specific status, such as the accumulation of reactive oxygen species or the reprogramming of hormone crosstalk pathways [33,34], influences host susceptibility and therefore entails changing scenarios for pathogens. However, how these differences may affect virus evolutionary strategies remains unknown.

Although the influence of host developmental stage during pathogen infection has gathered some attention, information is still lacking on how different host developmental stages might impose different selective environments for viruses, thus modulating their evolution. Most pathogen eco-evolutionary theories assume a link between virulence and transmission [35], as there should be a compromise between the duration of an infection (and therefore the time in which a pathogen can propagate and infect other hosts) and the damage that the pathogen causes to its host. Interestingly, it has been suggested that the virulence–transmission trade-off is affected by the host's life history [9,36,37]. Even though parasite-induced host mortality is the most used measure of virulence for horizontally transmitted parasites [38], there are pathogens that follow other evolutionary strategies to guarantee their reproduction and transmission without prematurely killing the host and, in consequence, themselves. This is the case of castrating parasites, which aim to diminish host fitness through interference with host fecundity. In fact, host castration has been argued to be an explicit evolutionary strategy of the parasite [39–41], because by castrating their hosts, pathogens can redirect host resources that were previously allocated for reproduction to their own reproduction and survival. Because of the reallocation of resources during development [42], even if viruses infect the same host genotype, the selective pressures imposed by the host may differ depending on the host age. And, therefore, adaptive mutations improving viral fitness in one developmental stage of the host may be selected against, or be neutral, in a different one.

The impact of the host developmental stage on its susceptibility to viruses is particularly relevant in the current situation, where climate change and anthropogenic activities are altering plant developmental patterns and timing. It has been reported that abiotic conditions associated with climate change can either induce or delay developmental processes and flowering in a genotype-dependent manner [43–46]. Flowering has been also described to be altered by the result of human activities such as urbanization [47,48] or biodiversity loss [49]. These changes in plant development will result in viruses facing host populations with altered levels of susceptibility. Understanding how these developmental alterations will impact the evolution of viruses is one key aspect to improve the management of viral diseases in a changing world.

Here we sought to better understand how host developmental stage influences virus evolution. We used *A. thaliana* (L.) Heynhold , a model organism in plant research [50], to evaluate the susceptibility to virus infection along three developmental stages: (i) vegetative juvenile stage, where plants allocate resources to increase their size and mass, (ii) bolting, an indicator of developmental stage transition [51], and (iii) flowering, where mature plants allocate resources to reproduction. We evaluated the host age effect on the susceptibility to infection for a collection of viruses from six different genera. After characterization of the host age-dependent susceptibility, we performed an evolution experiment using turnip mosaic virus (TuMV; species *Turnip mosaic virus*, genus *Potyvirus*, family Potyviridae) [52]. TuMV has a 9.5 kb positive single-stranded RNA genome that translates into a large polyprotein that is processed into 10 mature products plus a frameshift protein by three viral-encoded proteases [53]. TuMV infects over 300 plant species, though most belong to the Brassicaceae family [52–54]. We used two different TuMV isolates: one *naive* to *Arabidopsis* (hereafter referred as AS) and another previously evolved in and adapted to this host species (referred as DV). The resulting evolved lineages were phenotyped and sequenced to study the effects of host developmental stage on virus evolution. In summary, this work aims to (i) measure the impact of *Arabidopsis* developmental stage on susceptibility to virus infection and (ii) evaluate how virus evolution is affected by its host developmental stage.

## 2. Material and methods

### (a) Plant material and growth conditions

All experiments were performed in a growing chamber at 24°C during light periods and 20°C during dark, 45% relative humidity and 125 µmol m^−2^ s^−1^ light intensity (1 : 3 mixture of 450 nm blue and 670 nm purple LEDs). The photoperiod consisted of 16 h light/8 h dark for long-day conditions and 8 h light/16 h dark for short-day ones.

**Figure 1.**
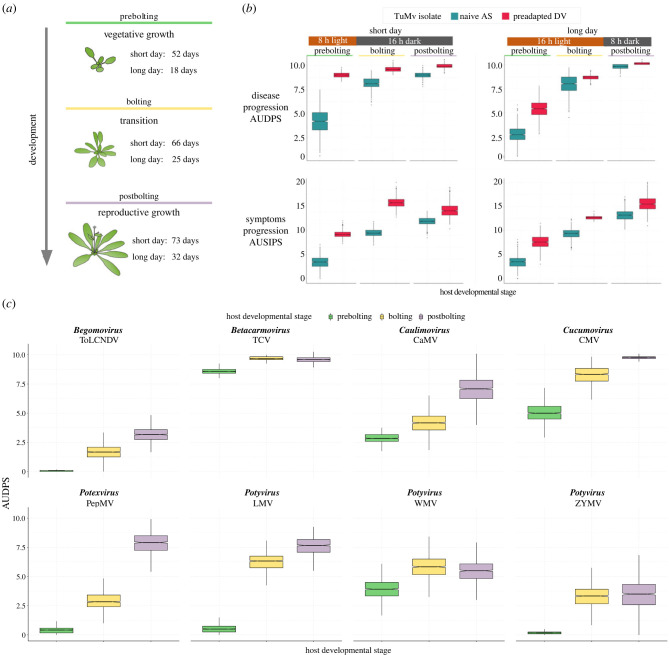
(*a*) Host developmental stages evaluated in this work and the corresponding number of days after sowing at which plants were inoculated under both photoperiod conditions. (*b*) Infection traits for turnip mosaic virus (TuMV) in short- and long-day photoperiod conditions. Box plot representation of progression of infectivity ((area under the disease progression stairs (AUDPS) values; upper row) and symptomatology progression (area under the intensity progression stairs (AUSIPS) values; lower row) for naive AS (blue) and preadapted DV (red) TuMV isolates in the three plant developmental stages evaluated. (*c*) Box plot representation of progression of infectivity (AUDPS values) for a set of viruses in the three plant developmental stages evaluated. For each virus, its genus is indicated. In the box plot, horizontal lines represent the median, and boxes represent the interquartile range (IQR), and error bars ±1.5 × IQR.

*Arabidopsis* plants of the Col-0 accession were used as hosts. Plants were inoculated at three different developmental stages: prebolting (juvenile), bolting (transition) and postbolting (mature) (figure 1*a*). In long-day conditions, these stages correspond to plants with an age of 18, 25 or 32 days after sowing, respectively. For short-day conditions, each stage corresponds to 52, 66 or 73 days after sowing, respectively. As bolting can be used as indicator of the vegetative to reproductive phase transition [51], each stage could be associated with different plant phases: vegetative growth, phase transition and reproductive growth. Following the growth stages described in [55], the inoculated plants also correspond to different principal growth stages (specific growth stages in parentheses): prebolting to stage 1 (1.06), bolting to stage 5 (5.10) and postbolting to stage 6 (6.00).

### (b) Viruses and experimental evolution

Inoculations were done using homogenized virus-infected tissue preserved at −80°C. The virus inoculum consisted of 100 mg of homogeneous N_2_-frozen infected tissue mixed with 1 ml of phosphate buffer and 10% carborundum (100 mg ml^−1^). For testing the susceptibility of *Arabidopsis* to different viruses, 12 plants per developmental stage were inoculated for each virus.

The following viruses were used: *Begomovirus* (tomato leaf curl New Delhi virus, ToLCNDV), *Betacarmovirus* (turnip crinkle virus, TCV), *Caulimovirus* (cauliflower mosaic virus, CaMV), *Cucumovirus* (CMV), *Potexvirus* (pepino mosaic virus, PepMV) and *Potyvirus* (lettuce mosaic virus, LMV; TuMV; watermelon mosaic virus, WMV; and zucchini yellow mosaic virus, ZYMV). Virus stocks were generated by harvesting and homogenizing infected tissue of *Arabidopsis* (CaMV, TCV and TuMV-DV), *Nicotiana benthamiana* Domin (CMV, LMV, PepMV, TuMV-AS and WMV) or *Chenopodium quinoa* Willdenow (ZYMV and ToLCNDV). The two isolates of TuMV used differ in their degree of adaptation to *Arabidopsis*: DV has an increased virulence and fixed mutations (CI/T1293I and VPg/N2039H) in comparation with AS. The naive AS isolate came from strain YC5 (GenBank, AF53055.2), originally obtained from calla lily (*Zantedeschia* sp.), which was cloned under the 35S promoter and NOS terminator, resulting in the p35STunos infectious clone [56]. The *Arabidopsis*-adapted DV isolate was obtained after experimentally evolving the AS isolate for 12 passages in prebolting Col-0 plants [3].

**Figure 2.**
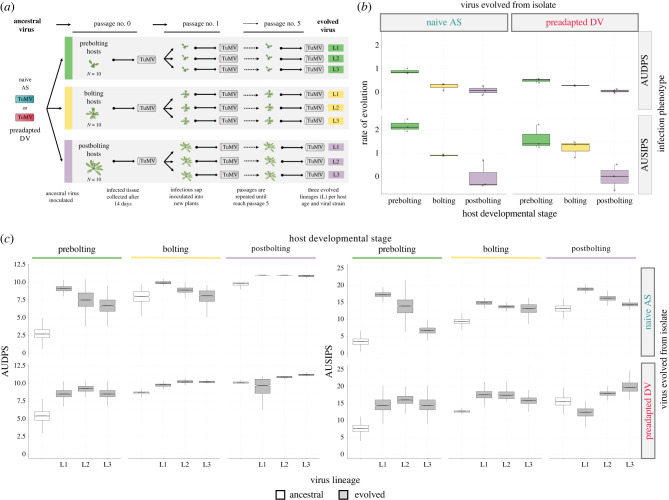
(*a*) Experimental evolution design. (*b*) Box plot representation of rates of phenotypic evolution for lineages evolved from the naive AS (left) and the preadapted DV (right) turnip mosaic virus (TuMV) isolates. Rates are calculated for both AUDPS (area under the disease progression stairs, upper row) and AUSIPS (area under the intensity progression stairs; lower row). (*c*) Box plot representation of evolved (grey) lineages' infection traits (AUDPSon the left; AUSIPS on the right) compared with their corresponding ancestral (white) viruses. Upper row shows the relative phenotype of viruses evolved from the naive AS isolate, while the lower row represents the values for viruses evolved from the preadapted DV isolate. In the box plot, horizontal lines represent the median, and boxes represent the interquartile range (IQR), and error bars ±1.5 × IQR.

For the evolution experiment, 10 plants were inoculated per combination of developmental stage and TuMV strain. Fourteen days post-inoculation (dpi), the symptomatic infected plants were collected, making a pool of infected tissue that was homogenized and used as inoculum to start a five-passages evolution. For each one of the three developmental stages, three independent lineages were established to serve as biological replicates of the evolutionary process (figure 2).

### (c) Infection characterization

Upon inoculation, plants were daily inspected for visual symptoms for 14 dpi and phenotyped following a discrete scale of symptoms severity from absence of symptoms (0) to full necrosis of the plant (5) (see Fig. 1 in [57]). The infectivity and severity of symptoms data collected during 14 dpi were used to calculate the area under the disease progression stairs (AUDPS) and intensity progression stairs (AUSIPS) values, respectively, as described in [58]. AUDPS and AUSIPS values were computed using the agricolae R package version 1.3.2 (https://myaseen208.github.io/agricolae/https://cran.r-project.org/package=agricolae) with R v.3.6.1 in RStudio v.1.2.1335.

### (d) Statistical analyses

A bootstrapping method described in [58] was used to estimate the confidence intervals (95% CIs) of AUDPS and AUSIPS.

AUDPS and AUSIPS evolution data were fitted to a generalized linear model (GLM) that incorporated a Gaussian probability distribution and an identity link function (according to the lowest Bayesian information criterion (BIC) value among a set of alternative models). Depending on the particular analyses, the model equations incorporated the developmental stage (vegetative, bolting and reproductive), photoperiod conditions (short and long days), virus isolate (AS and DV) or virus species as orthogonal factors, viral lineage as a nested factor within corresponding interactions among orthogonal factors being considered, and passage number as a covariable. For AUDPS, the significance of each factor was evaluated using a likelihood-ratio test that asymptotically follows a *χ*^2^ distribution. For AUSIPS, the significance of each factor was evaluated using an *F*-test with type III sum of squares. The magnitude of effects associated with each factor was evaluated using the $\eta_{p}^{2}$ statistic (proportion of total variability in the variables attributable to each factor in the model; conventionally, values of $\eta_{P}^{2}$ ≥ 0.15 are considered as large effects). The statistical power, 1−*β*, of each test was also computed. These analyses were performed with SPSS v.28.0.1.0 (IBM, Armonk, NY).

Rates of phenotypic evolution were evaluated by fitting the time-series data of AUDPS and AUSIPS to a first-order autoregressive integrated moving-average, ARIMA(1,0,0), model. The model equation fitted has the form:

2.1 $$A_{t}-\rho_{1}A_{t-1}=A_{0}+\nu t+\varepsilon_{t},$$

where *A_t_* represents the disease phenotypic values at passage *t*, *ρ*_1_ measures the degree of self-similarity in the time-series data (correlation between values at passages *t* and *t* – 1), *ε_t_* represents the sampling error at passage *t*, and *v* represents the dependency of *A* with passage number, that is, the rate of phenotypic evolution. In the case that measures taken at consecutive time points would be independent of each other, *ρ*_1_ = 0, and then equation (2.1) reduces to the simple linear regression model.

### (e) Sequencing of turnip mosaic virus

RNA was extracted from infected plant tissue using the NZY Total RNA Isolation Kit (NZYTech, Portugal) following the manufacturer's protocol and using a 30 mg quantity of plant material for extraction. The quality of the RNAs used to prepare RNA-seq libraries was checked with the Qubit RNA BR Assay Kit (Thermo Fisher, USA). SMAT libraries, Illumina sequencing (paired end, 150 bp) and quality check of the RNA-seq libraries were done by Novogene Europe. Nineteen bases from the 5′ end and 12 from the 3′ of the reads were trimmed with cutadapt version 2.10 [59]. Trimmed sequences were mapped with HiSAT2 version 2.1.0 [60] to the YC5 genome with a modified minimum score parameter (*L*, 0.0, −0.8) to allow more mismatches. Resulting SAM files were BAM-converted, sorted, indexed and analysed with SAMtools v.1.10 [61]. Single nucleotide polymorphism (SNP) calling was performed using bcftools v.1.6 by first using the mpileup subroutine [62].

## 3. Results

### (a) Host developmental stage increases susceptibility to turnip mosaic virus infection independently of the viral isolate

To test the effect of host developmental stage and photoperiod on disease traits (AUDPS and AUSIPS), we inoculated TuMV AS and DV isolates [3] in plants at the three aforementioned developmental stages. Both datasets were then fitted to the GLMs described in electronic supplementary material, table S1. First, regardless of the viral genotype and photoperiod conditions, disease progressed faster as plants became older (figure 1*b*, upper row; electronic supplementary material, table S1, main effect: *p* < 0.001, $\eta_{p}^{2}$ = 0.655 for AUDPS and *p* < 0.001, $\eta_{p}^{2}$ = 0.461 for AUSIPS). For instance, in long-day conditions, the nave AS isolate showed the lowest AUDPS in prebolting plants (mean ± 1 s.d.; 2.688 ± 0.015, which increased in bolting plants (7.930 ± 0.043) and further increased in postbolting plants (9.783 ± 0.053). The same trend was observed for plants infected with the preadapted DV isolate: this virus infected postbolting plants (10.112 ± 0.055) significantly better than plants at prebolting (5.331 ± 0.029) and bolting (8.672 ± 0.047) stages. Moreover, the results demonstrate differences between the two viral strains, where the preadapted DV virus infected significantly better than the naive one in all developmental stages (electronic supplementary material, table S1, main effect: *p* < 0.001, $\eta_{p}^{2}$ = 0.539 for AUDPS and *p* < 0.001, $\eta_{p}^{2}$ = 0.318 for AUSIPS). Furthermore, it is worth noting that differences in AUDPS between both isolates depended on the developmental stages (electronic supplementary material, table S1, interaction term: *p* < 0.001, $\eta_{p}^{2}$ = 0.984), being larger in prebolting than in the other two stages. Previous work [3] has described that, for TuMV inoculated into prebolting Col-0 plants under long-day conditions, infectivity progression correlates with virus accumulation and the severity of symptoms. Our results reinforce this observation, since the AUSIPS values completely mimic the results described for AUDPS (figure 1*b*, lower row).

Second, we tested whether the photoperiod conditions in which plants had been grown had any effect on the outcome of the experiment. In order to reach the three developmental stages evaluated in this work, different growing times were needed for each photoperiod condition. In the previous paragraph, we described the infection profile results obtained for a long-day condition photoperiod. For short-day conditions, we observed the same trend as described for the long photoperiod conditions: disease and symptomology progression were faster in late developmental stages (figure 1*b*). Hence, we rule out a major effect of the photoperiod in the observed results. Indeed, the GLM in electronic supplementary material, table S1 shows a net effect in AUDPS (*p* < 0.001, $\eta_{p}^{2}$ = 0.234) but not in AUSIPS, although the statistical power of this particular test was very low (1−*β* = 0.096), thus prompting caution against its robustness. The interaction between photoperiod and viral isolate was also significant for AUDPS (*p* = 0.031, $\eta_{p}^{2}$ = 0.835), though again the power was relatively low (1−*β* = 0.301).

### (b) The influence of host developmental stage on susceptibility to infection seems widespread among viruses

After showing the effect of host developmental stage on TuMV disease progression and severity, we sought to confirm whether this was a general feature or if it was dependent on the viral species being studied. To this end, we inoculated a total of eight viruses belonging to five different genera (see Material and methods for details). In these experiments, only AUDPS was evaluated. Data were fitted to the GLM shown in electronic supplementary material, table S2. Overall, the results reproduce those observed for TuMV: the older the *Arabidopsis* plants, the more susceptible they were to infection (figure 1*c*; electronic supplementary material, table S2, main effect: *p* < 0.001, $\eta_{p}^{2}$ = 0.461). Overall differences among viruses also exist (electronic supplementary material, table S2, main effect: *p* < 0.001, $\eta_{p}^{2}$ = 0.282), some being dependent on the precise developmental stage (electronic supplementary material, table S2, interaction term: *p* = 0.001), though the magnitude of this particular effect was moderate ($\eta_{p}^{2}$ = 0.124) In this sense, it is worth noticing that for some viruses, bolting and postbolting plants were significantly more susceptible compared with juvenile prebolting plants, but no significant differences existed between bolting and mature postbolting infected plants (figure 1*c*). This was the case for TCV (bolting, 9.668 ± 0.177; postbolting, 9.571 ± 0.241), WMV (bolting, 5.800 ± 1.041; postbolting, 5.374 ± 0.922) and ZYMV (bolting, 3.275 ± 0.990; postbolting, 3.497 ± 1.295).

### (c) Host developmental stage and virus evolutionary history shape the evolution of infection traits

After studying the role of host developmental stage on susceptibility to virus infection, we used the two aforementioned TuMV isolates to start a five-passage evolution experiment. Each isolate was evolved in *Arabidopsis* plants at each developmental stage maintained under long-day conditions (figure 2*a*). The results of the corresponding GLM are shown in electronic supplementary material, table S3. For both disease-related traits, developmental stage had a highly significant and large effect (AUDPS: *p* < 0.001, $\eta_{p}^{2}$ = 0.987; AUSIPS: *p* < 0.001, $\eta_{p}^{2}$ = 0.967), with mature postbolting plants having significantly larger values for both variables (*post hoc* sequential Bonferroni tests: *p* < 0.001), followed by bolting and juvenile prebolting, being equal for the two variables (*post hoc* sequential Bonferroni tests: *p* ≥ 0.455). Likewise, the viral isolate also had a major and highly significant effect in both traits consistent throughout the evolution experiment (AUDPS: *p* < 0.001, $\eta_{p}^{2}$ = 0.840; AUSIPS: *p* = 0.003, $\eta_{p}^{2}$ = 0.640), with DV being, overall, more virulent than AS. Moreover, these effects were not independent of each other: a significant interaction of a large magnitude between the two factors was also observed for AUDPS (*p* < 0.001, $\eta_{p}^{2}$ = 0.801) but not for AUSIPS. More relevant, the two disease variables significantly increased in value during the five-passage evolution experiment, showing evolutionary time also had an overall effect of large magnitude (AUDPS: *p* < 0.001, $\eta_{p}^{2}$ = 0.458; AUSIPS: *p* < 0.001, $\eta_{p}^{2}$ = 0.528). Finally, the effect of evolutionary time was affected by the developmental stage of the plants (AUDPS: *p* < 0.001, $\eta_{p}^{2}$ = 0.312; AUSIPS: *p* = 0.191, $\eta_{p}^{2}$ = 0.367), being larger for juvenile prebolting plants than for mature postbolting ones. No interaction between evolutionary time and viral isolate was detected (i.e. ancestral differences were maintained during the experiment) (electronic supplementary material, table S3: *p* = 0.078 for AUDPS and *p* = 0.400 for AUSIPS), nor higher-order interactions (electronic supplementary material, table S3: *p* = 0.063 for AUDPS and *p* = 0.338 for AUSIPS) or significant differences among independent lineages within each treatment (electronic supplementary material, table S3: *p* = 0.977 for AUDPS and *p* = 0.285 for AUSIPS).

The AUDPS and AUSIPS values of the evolved viruses were compared with the values obtained for their corresponding ancestral counterpart (figure 2*c*). We observed that the disease and symptomatology progression was faster in all the plants inoculated with evolved viruses in comparison with their antecessors. However, viruses evolved in juvenile prebolting hosts produced larger increases in disease severity (95% CI of ancestral viruses versus 95% CI of evolved viruses), and this increase was even larger for the naive AS isolate ([2.293–2.410] versus [7.678–7.753]) than for the preadapted DV one ([5.766–5.877] versus [8.226–8.283]), when comparing with bolting ([7.873–7.978] versus [8.642–8.699]; [8.690–8.721] versus [9.863–9.884]) or postbolting hosts ([9.684–9.711] versus [10.588–10.633]; [10.094–10.106] versus [10.558–10.601]).

At the end of the evolution experiment, the rate of phenotypic evolution for the two disease-related traits AUDPS and AUSIPS was evaluated using the ARIMA model shown in equation (2.1). Our results show that the younger the host, the faster the evolution of both disease traits (figure 2*b*). This happened in hosts infected with the naive AS isolate, both for disease progression AUDPS, where the evolutionary rate was higher in prebolting (average across lineages: 0.881) than in bolting (versus 0.244; *post hoc* sequential Bonferroni test *p* < 0.001) and postbolting (versus 0.066; *post hoc* sequential Bonferroni test *p* < 0.001) plants, and for symptom progression AUSIPS: the evolutionary rate was higher in prebolting (2.166) than in bolting (versus 0.912; *post hoc* sequential Bonferroni test *p* < 0.001) and postbolting (versus −0.012; *post hoc* sequential Bonferroni test *p* < 0.001) plants. A qualitatively similar pattern was observed for the preadapted DV isolate: the rate of AUDPS evolution was higher in prebolting (0.496) than in bolting (versus 0.276; *post hoc* sequential Bonferroni test *p* = 0.029) and postbolting (versus 0.048; *post hoc* sequential Bonferroni test *p* = 0.007) plants; that for AUSIPS was also higher in prebolting (1.626) than in bolting (versus 1.216; *post hoc* sequential Bonferroni test *p* = 0.018) and postbolting (versus −0.061; *post hoc* sequential Bonferroni test *p* < 0.001) plants. These results are consistent with those obtained with the GLM analysis (see above).

### (d) Genomic changes in evolved viruses dependent on the viral isolate and the host age

**Figure 3.**
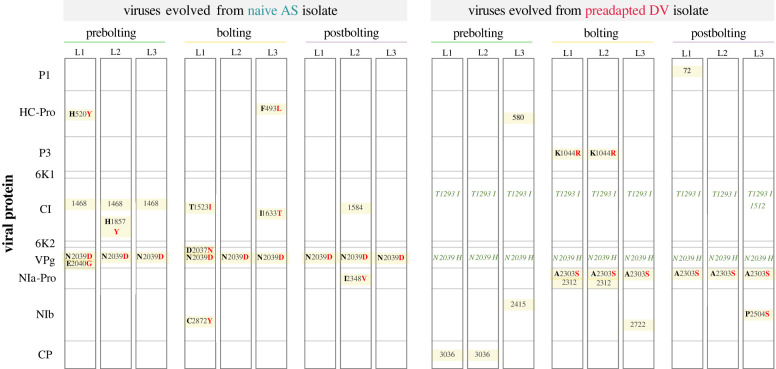
Mutations observed on every evolved lineage. The ancestral isolates, plant developmental stage and lineage are indicated above. Rectangles are proportional to the size of the cistron indicated in the first column. Synonymous mutations are indicated with the number of the nucleotide position in the polyprotein. Non-synonymous mutations are indicated with the ancestral amino acid in black and the mutated one in red. Non-synonymous mutations that were already present in the preadapted DV isolate are indicated in green.

The genomes of the evolved viruses were sequenced to identify mutations that arose during the experimental evolution process (figure 3). All lineages evolved from the naive AS isolate had the same non-synonymous mutation in the VPg/N2039D, regardless of the developmental stage of the host in which they were evolved. Interestingly, this mutation affects the same amino acid residue as mutation VPg/N2039H, which was already present in the preadapted DV isolate. Therefore, mutations at amino acid 2039 of VPg might be involved in specific adaptation to *Arabidopsis*. However, these two mutations likely have different effects in VPg structure and function: whereas N2039D replaces a polar amino acid by a negatively charged residue, N2039H replacement implies the presence of a bulky and positively charged residue. Other non-synonymous mutations affecting HC-Pro, CI or NIb have been observed in other lineages, yet none was shared. By contrast, the three AS-derived lineages evolved in prebolting hosts shared a non-synonymous mutation in CI (amino acid residue 1468).

For lineages evolved from the preadapted DV isolate, the mutation pattern was markedly different: no additional mutations were found in the *VPg* gene. For the three lineages evolved in prebolting plants, all the mutations were synonymous. By contrast, for lineages evolved in bolting and postbolting plants, a non-synonymous mutation NIa-Pro/A2030S (small non-polar to polar change) was shared by all six independent lineages. In addition, two lineages evolved in bolting plants also shared mutation in P3/K1044R (conservative change of long positively charged side chains).

## 4. Discussion

Host developmental stage constitutes an often-overlooked factor when studying plant–virus interactions and the outcome of an infection. In plants, there is evidence of the reassignment of resources between flowering and defence [30]. Classic life-history evolution theories predict that selection on any trait weakens as organisms age [63,64]. According to this assumption, fitness-related traits such as immune defence would be under weaker selection in older hosts; thus, a decline in host resistance to pathogens over time is expected. This assumption seems to hold for our pathosystem, *A. thaliana*–TuMV.

Even though a correlation between ageing and increased host resistance to infection seems to be common in plants, the ARR defence mechanism which explains this phenomenon has mainly been described for plant–bacteria interactions [16–18]. Our results with viruses are somehow at odds. We are somehow still blind on this matter as there are not many studies highlighting the infection dynamics and mechanisms connecting host developmental stage and virus infection. While there are examples of aged plants being more resistant to viral infections than juveniles, others show the opposite trend. García-Ruiz & Murphy [15] showed in the *Capsicum annuum*–CMV pathosystem that severity of symptoms and viral accumulation in non-inoculated leaves reached higher levels in juveniles than in mature plants. Levy & Lapidot [65] showed in the *Solanum lycopersicum*–tomato yellow leaf curl virus pathosystem that infected juvenile plants had significantly lower yield compared with mature plants. In sharp contrast yet in line with our observations, a recent study with the *Arabidopsis*–tomato spotted wilt virus (TSWV) and *Arabidopsis*–CMV pathosystems illustrates a developmentally regulated increase in susceptibility as plants become older under short-day photoperiod [23]. Whether the existing ARR response described for other pathogens plays a role in virus infection remains unclear.

Under our experimental conditions, *Arabidopsis* reproduces only once before dying. Hence, it makes sense that our results lean towards a survival strategy that prioritizes reproduction over defences. In nature, however, two different cohorts of *Arabidopsis* can be found, depending on the timing of germination and the state in which the plant overwinters [66]. In general, it is commonly accepted that the winter cohort, which germinates in autumn and overwinters as a vegetative rosette until its flowering in spring, is the most widespread one [67]. Under these conditions, where plants need to survive all winter in a phase of vegetative growth, it might be beneficial to invest in defence in order to survive until the next spring. By contrast, for the summer cohort, which germinates in late spring/early summer and produces mature seeds by late summer, it might be beneficial to invest in fast development, as in our experimental design.

The relationship between defence and host developmental stage may depend on the host and/or pathogen genetics and the interplays between the two. Huang *et al*. [23] observed that host developmental-stage-dependent susceptibility does not occur in some host species such as tomato, pepper or *N. benthamiana* (all Solanaceae) but it does occur in *Arabidopsis* (Brassicaceae). It needs to be further studied whether developmental-stage-dependent susceptibility is a common phenomenon in other plant families. In their study, Huang *et al*. [23] infected *Arabidopsis* with CMV and TSWV and demonstrated that these viruses infected mature plants better than juvenile ones. Our results with a set of viruses belonging to different genera align well with their findings. However, for some viruses, like TCV, WMV and ZYMV, we did not observe a significant difference between disease progression values in bolting and postbolting plants. Hence, whether this is a universal property of the way *Arabidopsis* interacts with viral pathogens or just a spurious consequence of the chosen viruses and limited sample size (*n* = 11 viral species in total) needs to be further explored. Indeed, Pagán *et al*. [32] proved that CMV virulence depended on the infection of specific *Arabidopsis* genotypes, which contributes to the idea that the infection outcome directly depends on the interaction between specific hosts and viruses. Furthermore, to explore the effect of fitness differences among genetic variants of the same virus, we compared the results obtained for two isolates of TuMV. We observed that both isolates displayed the same host developmental stage dependence, infecting mature postbolting hosts better, although the room for improvement of infection traits for the poorly adapted isolate was larger, as shown before for TuMV [58] and for bacteriophages used in phage therapy preadapted to *Pseudomonas aeruginosa* [68].

Our study also suggests, at least for TuMV, that prebolting plants are generally more susceptible to virus infection in short days than in long ones. This observation is in line with the results reported in [69], showing that reactive oxygen species (key elements for the successful activation of plant immune responses) in tobacco leaves kept in short days reached higher levels than when maintained under long days. Despite photoperiod-dependent susceptibility, the observation of later developmental stages being more susceptible to viruses was consistent in both short- and long-day conditions.

Our results indicate that host developmental stage can be an important factor modulating virus evolution. We performed a short five-passage evolution experiment, which was sufficient to see changes in evolved RNA viruses [70–72]. In our experimental pathosystem we observed that infection proceeds faster in mature postbolting hosts, indicating they are more susceptible. However, phenotypic rates of evolution are slower in the mature postbolting hosts than in the juvenile prebolting ones. This can be easily explained by differences in selective pressures: while postbolting hosts are permissive and represent a weak selective environment, prebolting ones are more restrictive to infection and represent a harsh selective environment. Thus, adaptive mutations in the latter might provide a disproportionally larger fitness benefit than in the former. From this, it is tempting to speculate whether certain developmental stages might select for viruses that specialize in infecting that particular stage or, by contrast, other more complex yet interesting possibilities exist: e.g. viruses selected in the more restrictive juvenile prebolting plants would behave as generalists infecting all developmental stages equally well whereas viruses selected in the more permissive mature postbolting ones would act as host developmental stage specialists. This is currently being tested in the laboratory.

Depending on the developmental stage when plants were infected, the differential host–virus interactions caused viruses to face different evolutionary constraints, which gave rise to different mutational spectra. After the five-passage experimental evolution, viral lineages evolved from the preadapted DV in bolting and postbolting hosts selected for the same mutation in the *NIa-Pro* cistron (A2303S). This suggests that this viral protein may play a key role during infection in these older stages but not in the juvenile prebolting one. Notably, DV-derived lineages evolved in juvenile prebolting plants show no fixed non-synonymous mutations. This apparent genetic stability could be partially explained by the past evolutionary history of the DV isolate: since it was already preadapted for 10 passages in prebolting conditions, we can confidently assume that specific adaptations to this developmental stage had already taken place. The presence of the same synonymous mutation in position 1468 of the CI protein in all lineages evolved from the naive AS isolate in prebolting plants suggests that the selective pressure imposed by the host may be different depending on the host developmental stage. It is important to note that certain selective pressures may still be independent of the host developmental stage, as suggested by certain mutational events: (i) lineages evolved from the naive AS isolate in bolting and postbolting hosts have some different mutations in CI. (ii) Likewise, the preadapted DV isolate fixed mutation CI/T1293I during its original evolution in prebolting plants, suggesting that this protein may be targeted independently of the host developmental stage. This mutation has been retained in all DV-derived lineages irrespective of their host developmental stage. (iii) During its original evolution, DV also acquired mutation VPg/N2039H, which has also been conserved in all its derived lineages. Interestingly, all lineages evolved from the naive AS isolate had fixed mutation VPg/N2039D in the same position. The selection on VPg mutants at position N2039 seems related with the functions of this protein: virus movement, genome replication and suppression of host antiviral RNA silencing [53]. Previous evolutionary experiments from our group have also described mutations in the same region of VPg on evolved viruses with increased virulence, independently of the *Arabidopsis* genotype, its susceptibility to infection or the environmental conditions [70,73]. Similarly, mutations in this protein increased the fitness of TuMV in *Arabidopsis* plants with knock-outs in the *eIF(iso)4E* and *eIF(iso)4G* genes [74] as well as the virulence of potato virus Y in resistant pepper plants [75].

## 5. Conclusion

In this work, we aimed to study the impact of host developmental stage on plants' susceptibility to viruses. We observed a positive correlation between host developmental stage and its susceptibility to multiple viruses belonging to different genera. Focusing on a potyvirus species, TuMV, we observed that this correlation occurs independently of the degree of adaptation of the viral isolate or the photoperiod the plant host is exposed to. We also studied the impact of host developmental stage on virus evolution. First, we reported an increase in disease and symptomatology progression when comparing evolved viruses with their ancestral ones. This increase was more prominent the younger the host the virus was evolved in. Second, we show that host developmental stage conditioned the spectra of mutations that appeared in the evolved viral lineages.

This work does not provide any insight into the molecular mechanisms behind these observations. A better understanding of the host response will also contribute to identification of the selective pressures that viruses face during evolution in hosts at different developmental stages. A follow-up paper will focus on the host transcriptomic and hormonal signalling responses to TuMV infection at each developmental stage. With this approach, we will try to understand which host responses are responsible for the observed differences in susceptibility. It is important to note that our research was done using only one *Arabidopsis* genotype. Considering that most plant–virus interactions are dependent on the host genotype, it will be of great interest to study how common this phenomenon is among different plant species and strains. Furthermore, even if the phenomenon is common among diverse plant genotypes the underlying mechanism could be genotype-specific.

Overall, this work confirms that late plant host developmental stages may have an increased susceptibility to viruses and describes for the first time to our knowledge its impact on virus evolution. The results of this study contribute to better understanding on how the host developmental stage shapes virus evolution, which is key to manage current and future viral diseases.

## Data Availability

All data generated in this work can be access in Zenodo under: https://doi.org/10.5281/zenodo.6973930 [76]. The data are also provided in the electronic supplementary material [77].
